# From the Cochrane Library: Interventions for Pemphigus Vulgaris and Pemphigus Foliaceus

**DOI:** 10.2196/46812

**Published:** 2023-12-15

**Authors:** Ramiro Rodriguez, Torunn E Sivesind, Dedee Murrell, Robert P Dellavalle

**Affiliations:** 1 Department of Dermatology University of Colorado Anschutz Medical Campus Aurora, CO United States; 2 Department of Dermatology St George Hospital Sydney Australia; 3 Dermatology Service Rocky Mountain Regional VA Medical Center Eastern Colorado Health Care System Aurora, CO United States; 4 Department of Epidemiology Colorado School of Public Health University of Colorado Anschutz Medical Campus Aurora, CO United States

**Keywords:** pemphigus vulgaris, pemphigus foliaceus, Cochrane, rituximab, desmoglein, vesiculobullous, immunoglobulin, corticosteroids, skin, dermatology, systematic review

## Introduction

Cochrane systematic reviews are rigorous in methodology and contribute to our understanding of evidence-based treatments of diseases. Among these diseases is pemphigus, a group of acquired autoimmune vesiculobullous diseases (pemphigus vulgaris [PV], pemphigus foliaceus [PF], and pemphigus paraneoplastic [PNP]), characterized by B-cell–mediated immunoglobulin G antibodies against desmogleins 1 and 3. Cutaneous bullae cause loss of barrier function and pain, dehydration, superimposed infections, and psychological distress. Due to a lack of expert consensus and the poor efficacy of previous therapies, a Cochrane systematic review of randomized controlled trials (RCTs) sought to define the best treatment for pemphigus [1]. Here, we highlight takeaways from the review [1] and discuss advances in therapy. PNP was excluded from the review due to its rarity and because its management depends on the underlying malignancy.

## Methods

A total of 11 RCTs were analyzed to assess efficacy and safety among treatments for PV and PF. The primary outcomes were death and disease remission, with secondary outcomes including disease severity indexes, time to disease control, cumulative glucocorticoid dose, serologic markers, and the proportion of patients achieving disease control and relapse. The RCTs used various combinations and doses of steroid-sparing agents with or without corticosteroids. We contrasted therapies, outcomes, and comparison effect size and conducted 4 meta-analyses.

## Results

A recent (2021) network meta-analysis [2] found rituximab (Table 1), a CD20 B-cell–depleting therapy, as the most effective therapy for key outcomes like disease relapse, withdrawal from adverse events, remission, and cumulative glucocorticoid dose. The right-most column of Table 1 contrasts therapies relative to rituximab among the 4 key outcomes evaluated. Although the included trials [2] risked bias due to inadequate allocation concealment and lack of participant, personnel, and outcome blinding, the results align with emerging expert consensus and other important clinical trials [3] directly comparing rituximab to other therapies like mycophenolate.

**Table 1:** Summary of interventions, outcomes assessed, and effect size from 2009 (left) and 2021 (right).

2009								2021			
Intervention			Effect size: RR^a^ (95% Cl)		Outcomes		Effect size: pooled OR^b^ (95% CI)			Rituximab vs intervention	
**Prednisolone (1mg/kg vs 0.5 mg/kg)**											Steroid alone
	1	Not estimable		Disease control		—^c^					
	2	0.7 (0.43 to 1.14)		Relapse		0.38 (0.12 to 1.15)					
	3	Not estimable		Withdrawal due to adverse event		0.05 (0 to 0.083)					
**Pulsed oral dexamethasone vs placebo**											Steroid alone
	1	1.91 (0.68 to 5.33)		Relapse (after discontinuing or stopping)		—					
	2	2.45 (0.31 to 19.74)		Withdrawal due to adverse event		—					
**Azathioprine vs glucocorticoid (prednisolone) alone**											
	1	1.04 (0.8 to 1.36)		Remission		14.45 (4.71 to 43.68)			Steroid alone		
	2	–3.91 (–6.71 to –1.12)		Cumulative glucocorticoid dose		–11.10 (–14.08 to –9.57)			Steroid alone		
	3	2 (0.19 to 20.9)		Withdrawal due to adverse event		0.02 (0 to 0.56)			Azathioprine		
**Cyclophosphamide vs glucocorticoid (prednisone/prednisolone) alone**											Azathioprine
	1	0.96 (0.71 to 1.28)		Remission		10.10 (2.67 to 38.23)					
	2	0 (0)		Disease control		—					
	3	0.5 (0.05 to 4.67)		Relapse		0.60 (0.10 to 3.63)					
	4	–3.35 (–6.14 to –0.56)		Cumulative glucocorticoid dose		­–8.79 (–11.60 to –5.98)					
	5	0.33 (0.01 to 7.87)		Withdrawal due to adverse events		—					
**Cyclosporine vs glucocorticoid (prednisone/methylprednisolone) alone**											Cyclophosphamide
	1	0 (0)		Remission		9.59 (2.42 to 37.96)					
	2	1.06 (0.86 to 1.32)		Disease control		—					
	3	0.92 (0.23 to 3.65)		Relapse		0.42 (0.08 to 2.28)					
	4	–0.05 ( –0.18 to 0.081)		Cumulative glucocorticoid dose		–9.36 (–12.16 to –6.55)					
	5	0 (0)		Withdrawal due to adverse event		0.10 (0 to 4.20)					
**Dapsone vs placebo**											Cyclophosphamide
	1	1.85 (0.61 to 5.63)		Remission (<7.5 mg prednisone) at 12 months		—					
	2	0.37 (0.05 to 2.95)		Withdrawal due to adverse event		—					
**Mycophenolate vs glucocorticoid (prednisolone) alone**											Dexamethasone-cyclophosphamide (6 and 12 months)
	1	0.91 (0.67 to 1.24)		Remission		47.11 (4.99 to 445.07), 6 months					
	2	–1.83 (–4.94 to 1.28)		Cumulative glucocorticoid dose		—					
	3	1.0 (0.07 to 15.26)		Withdrawal due to adverse events		0.06 (0 to 7.06), 6 months					
**Plasma-exchange vs control**											Dexamethasone-cyclophosphamide (6 and 12 months)
	1	7.43 (0.43 to 129.55)		Death		—					
	2	1.12 (0.70 to 1.78)		Disease control (study definition involving relative healing time)		—					
	3	44.38 (–222.43 to 311.19)		Reduction antibody titer (baseline to end protocol, mean difference)		—					
	4	7.2 (0.42 to 124.08)		Withdrawal due to adverse events		—					
**Azathioprine vs cyclophosphamide**											
	1	1.09 (0.82 to 1.44)		Remission		5.48 (0.71 to 42.02), 12 months			Dexamethasone-cyclophosphamide (6 and 12 months)		
	2	1.8 (0.89 to 3.64)		Disease control (healing of >50% of lesions and/or occurrence of <5 blisters/month)		—			Dexamethasone-cyclophosphamide (6 and 12 months)		
	3	1.0 (0.53 to 1.88)		Relapse		0.67 (0.04 to 11.13)			Dexamethasone-cyclophosphamide (6 and 12 months)		
	4	1.0 (0.53 to 1.88)		Relapse		0.063 (0.12 to 3.47)			Mycophenolate		
	5	–5.64 (–1.04 to –0.79)		Cumulative glucocorticoid dose		—			Mycophenolate		
	6	3.91 (0.45 to 33.66)		Withdrawal due to adverse events		0.05 (0 to 1.18)			Mycophenolate		
**Azathioprine vs mycophenolate**											Mycophenolate
	1	1.14 (0.85 to 1.53)		Remission		10.80 (3.07 to 38.05)					
	2	0.72 (0.52 to 0.99)		Disease control		—					
	3	–2.07 (–3.54 to –0.60)		Cumulative glucocorticoid dose		–11.10 (–13.70 to –8.49)					
	4	3.01 (0.48 to 18.97)		Withdrawal due to adverse events		—					
**Cyclophosphamide vs cyclosporine**											
	1	0 (0)		Remission (<10 mg prednisone equivalent) at 5 years		—			Mycophenolate		
	2	0 (0)		Disease control		—			Mycophenolate		
	3	0.4 (0.04 to 3.66)		Relapse		0.81 (0.05 to 13.72)			Cyclosporine		
	4	0 (0)		Withdrawal due to adverse events		0.04 (0 to 5.92)			Cyclosporine		
**Cyclophosphamide vs mycophenolate**											Cyclosporine
	1	1.05 (0.76 to 1.44)		Remission		11.96 (1.92 to 74.49)					
	2	–1.52 (–2.98 to –0.056)		Cumulative glucocorticoid dose		–11.77 (–14.04 to 9.51)					
	3	0.33 (0.01 to 7.87)		Withdrawal due to adverse events		—					
Topical epidermal growth factor vs placebo			2.35 (1.62 to 3.41)		Time to control (hazard ratio)		—			Cyclosporine	
Traditional Chinese Medicine			0.75 (–1.12 to 2.62)		Antibody titer		—			Cyclosporine	

^a^RR: relative risk.

^b^OR: odds ratio.

^c^The 2021 network review assessed withdrawal due to adverse events, remission, relapse, and cumulative glucocorticoid dose. Other measures were not available.

Induction dosing for rituximab was two 1 g intravenous infusions 2 weeks apart followed by a 6-month prednisone taper of 1 mg/kg/day. Additionally, 2 novel higher-affinity CD20-blocking agents, ofatumumab and veltuzumab, demonstrated efficacy in isolated cases of rituximab-resistant pemphigus. Ofatumumab and veltuzumab are not used for pemphigus outside of clinical trials and for compassionate use. In addition, trials are underway for other immunotherapies targeting the fragment crystallizable region, B-cell–activating factor, and Bruton tyrosine kinase [4]. The meta-analyses revealed that some interventions were superior for certain outcomes: improved disease remission with mycophenolate relative to azathioprine, a steroid-sparing effect with azathioprine and cyclophosphamide, and a decreased time to erosion control with topical epidermal growth factor (Table 2). At the time of the 2009 study, systematic analysis including rituximab and clinical trials including intravenous immunoglobulin were ongoing [5].

**Table 2:** Summary of conclusive secondary outcomes (2009).

Therapeutic	Secondary outcome
Mycophenolate mofetil	Improved disease control compared to azathioprine (RR^a^ 0.72, 95% CI 0.52 to 0.99; NNT^b^ 3.7)
Azathioprine	Decreased the cumulative glucocorticoid dose (MWD^c^ –3919 mg prednisolone, 95% CI –6712 to –1126)
Cyclophosphamide	Deceased the cumulative glucocorticoid dose compared to prednisolone alone (MWD –3355 mg prednisolone, 95% CI –6144 to –566)
Topical epidermal growth factor	Decreased time to erosion healing compared to the control intervention (HR^d^ 2.35, 95% CI 1.62-3.41)

^a^RR: relative risk.

^b^NNT: number needed to treat.

^c^MWD: difference in means.

^d^HR: hazard ratio.

## Discussion

With an increasing understanding of the immune system, B-cell physiology, and the pathogenesis of pemphigus, therapies continue to emerge, making previous therapies obsolete. Here, we placed important Cochrane review findings in the context of recent advancements in the treatment of pemphigus. Further studies are needed to determine therapeutic regimens, safety, and efficacy of novel medical therapies for pemphigus.

## Abbreviations

PF: pemphigus foliaceus
PNP: pemphigus paraneoplastic
PV: pemphigus vulgaris
RCT: randomized controlled trial
